# Differentiation of *Mycobacterium tuberculosis* complex from non-tubercular mycobacteria by nested multiplex PCR targeting *IS6110*, *MTP40* and *32kD alpha antigen* encoding gene fragments

**DOI:** 10.1186/s12879-016-1450-1

**Published:** 2016-03-12

**Authors:** Pallavi Sinha, Anamika Gupta, Pradyot Prakash, Shampa Anupurba, Rajneesh Tripathi, G. N. Srivastava

**Affiliations:** Department of Microbiology, Institute of Medical Sciences, Banaras Hindu University, 221 005 Varanasi, India; Departmrnt of TB and Respiratory Diseases, Institute of Medical Sciences, Banaras Hindu University, Varanasi, India

**Keywords:** *Mycobacterium tuberculosis* complex, Non-tubercular mycobacteria, Nested multiplex PCR, Composite reference standard

## Abstract

**Background:**

Control of the global burden of tuberculosis is obstructed due to lack of simple, rapid and cost effective diagnostic techniques that can be used in resource poor-settings.

To facilitate the early diagnosis of TB directly from clinical specimens, we have standardized and validated the use of nested multiplex PCR, targeting gene fragments *IS6110*, *MTP40* and *32kD* α*-antigen* encoding genes specific for *Mycobacterium tuberculosis* complex and non-tubercular mycobacteria (NTM), in comparison to smear microscopy, solid culture and single step multiplex PCR. The results were evaluated in comparison to a composite reference standard (CRS) comprising of microbiological results (smear and culture), clinical, radiological and cytopathological findings, clinical treatment and response to anti-tubercular therapy.

**Methods:**

The nested multiplex PCR (nMPCR) assay was evaluated to test its utility in 600 (535 pulmonary and 65 extra-pulmonary specimens) clinically suspected TB cases. All specimens were processed for smear, culture, single step multiplex PCR and nested multiplex PCR testing.

**Results:**

Out of 535 screened pulmonary and 65 extra-pulmonary specimens, 329 (61.5 %) and 19 (29.2 %) cases were culture positive for *M. tuberculosis*. Based on CRS, 450 patients had “clinical TB” (definitive-TB, probable-TB and possible-TB). Remaining 150 were confirmed “non-TB” cases. For culture, the sensitivity was low, 79.3 % for pulmonary and 54.3 % for extra-pulmonary cases. The sensitivity and specificity results for nMPCR test were evaluated taken composite reference standard as a gold standard. The sensitivity of the nMPCR assay was 97.1 % for pulmonary and 91.4 % for extra-pulmonary TB cases with specificity of 100 % and 93.3 % respectively.

**Conclusion:**

Nested multiplex PCR using three gene primers is a rapid, reliable and highly sensitive and specific diagnostic technique for the detection and differentiation of *M. tuberculosis* complex from NTM genome and will be useful in diagnosing paucibacillary samples. Nested multiplex PCR assay was found to be better than single step multiplex PCR for assessing the diagnosis of TB.

**Electronic supplementary material:**

The online version of this article (doi:10.1186/s12879-016-1450-1) contains supplementary material, which is available to authorized users.

## Background

Tuberculosis (TB), caused by *Mycobacterium tuberculosis* complex (MTBC), still remains the major killer disease worldwide, especially in developing countries in spite of considerable progress in diagnosis and treatment [1]. *Mycobacterium tuberculosis* complex comprises of *Mycobacterium tuberculosis* (MTB)*, M. africanum, M. canettii, M. bovis, M. microti, M. orygis, M. caprae, M. pinnipedii, M. suricattae* and recently recognized *M. mungi* [2]*.* According to WHO there were approximately 9.0 million new cases and 1.5 million deaths globally in 2014. India contributed the highest number of new cases of TB, accounting for 24 % of the global burden [1]. Extra-pulmonary tuberculosis (EPTB) contributes about 15–20 % of the total cases of tuberculosis worldwide [1]. A major obstacle to the diagnosis of EPTB is the atypical presentation, often simulating neoplasia and/or inflammatory disorders.

The non-tuberculous mycobacterial (NTM) infections have also increased in many regions of the world along with MTBC infections and much of this increase in the burden of TB concurred with human immune deficiency virus (HIV) infection in patients [3, 4]. The species of NTM associated with human disease are: *M. avium*, *M. intracellulare*, *M. kansasii, M. fortuitum, M. chelonae, M. szulgai, M. paratuberculosis, M. scrofulaceum* etc. Most of the incidence of NTM infections has been reported from TB non-endemic countries and rarely from TB endemic countries because the chances of missing NTM infection are higher in TB endemic countries [5, 6].

The current standard of care for diagnostic techniques does not include bacterial characterization. Consequently, some NTM cases with positive smears will continue to be misclassified as MTB and receive chemotherapy commonly used for tuberculosis due to which some of the NTM strains may be resistant. Hence, majority of NTM infections will remain undetected. In addition, cases of mixed infection have also been reported [4, 7].

The conventional methods such as smear microscopy has low sensitivity and specificity and culture is time consuming (6–8 weeks) because of the slow growth rate of TB bacilli [7–9]. To overcome these problems, nucleic acid amplification test (NAAT) has been used for diagnosis of pulmonary tuberculosis (PTB) and extra-pulmonary tuberculosis [10]. In case of extra-pulmonary specimens there is a lack of sensitivity of conventional PCR techniques as they are mostly paucibacillary in nature. Another major limitation of single step PCR for pulmonary and extra-pulmonary specimens is the presence of PCR inhibitors that inhibit the amplification based techniques. Therefore, a two step process is necessary to eliminate/dilute the inhibitors present in the clinical specimens.

The target sequences *IS6110* and *MTP40* have been used in multiplex PCR for the diagnosis of pulmonary and extra-pulmonary TB which increases the sensitivity for detection [11–15]. One study has reported a robust, reproducible and uniform nested PCR (nPCR) protocol for the removal of PCR inhibitors, but nested PCR lacks the specificity by using single target sequence [16]. Some studies have also compared nucleic acid amplification test with composite reference standard (CRS) and culture [17–19], but these techniques could not differentiate MTBC from NTM. Therefore, we have used nested multiplex PCR (nMPCR) assay and compared it with composite reference standard and single step multiplex PCR (mPCR) assay to evaluate the true diagnostic potential of the nMPCR assay for pulmonary and extra-pulmonary TB. The CRS for this study comprised of signs, symptoms, radiological scans, cytolopathology, microbiological results (smear and LJ culture), previous and family history and response to anti-tubercular therapy (ATT).

## Methods

### Study design

This study was conducted during the period May 2012 - February 2014 at the Department of Microbiology, Institute of Medical Sciences, Banaras Hindu University, Varanasi, India. The patients registered in this study were attending either outpatient department or were admitted in the ward, Department of Chest and Respiratory diseases, Sir Sunder Lal Hospital, Institute of Medical Sciences, Banaras Hindu University.

### Specimen collection

A total of 600 clinical specimens were studied, including 535 pulmonary [523 sputum and 12 Bronchoalveolar Lavage (BAL)] and 65 extra-pulmonary specimens from outpatients and inpatients of a tertiary care centre of Banaras Hindu University, Varanasi. The 65 extra-pulmonary specimens included 15 sterile body fluids (5 pleural fluid, 9 cerebrospinal fluid (CSF) and one blood from bone marrow), 15 pus samples, 20 urine samples and 15 fine needle aspirates (FNAs). Each sample was divided into two parts. First part was used for microbiological investigations (microscopy and culture) and second part was processed for PCR.

### Clinical assessment of patients

The patients were categorized on the basis of composite reference standard criteria.

(A) Definitive TB groups: AFB smear positive and culture positive (S + C+) and AFB smear negative and culture positive (S-C+),
(B) Probable TB groups: Patients’ selection with AFB smear positive and culture negative (S + C-) but showing clinical symptoms, chest X-ray highly indicative of TB and/or cytology suggestive of TB,
(C) Possible TB groups: smear negative and culture negative (S-C-) and only clinical signs and/or symptoms suggestive of TB; patients response to ATT and
(D) Non-TB groups: microbiological tests for TB were negative and patient improved without taking ATT but two specimens were obtained from patients with lymphadenopathy and metastatic carcinoma.

### Sample preparation

Sputum, pus and BAL (bronchoalveolar Lavage) samples were processed using Petroff’s method (4 % NaOH) [20]. One loopful (approximately 0.1–0.15 ml) of sterile body fluids like pleural fluid, CSF and blood from bone marrow were inoculated directly on Lowenstein Jensen (LJ) medium. If the specimen volume was more than 10 ml, concentration by centrifugation was done at about 3000–3500 × g for 15–20 min [21, 22]. FNAC samples were collected aseptically, dispensed in 200 μl phosphate buffer saline and one loopful was directly inoculated on LJ medium. Urine samples were concentrated by centrifugation at 3000 × g for 15 min followed by decontamination in a similar manner as sputum [22].

### Microscopy and culture

All pulmonary and extra pulmonary clinical specimens and the sediments were subjected to smear examination through the standard Ziehl Neelsen’s staining method and were cultured onto a pair of Lowenstein-Jensen slants [23]. The LJ slants were incubated at 37 °C and inspected weekly for mycobacterial growth for a period of 8 weeks. Cultures grown were identified by standard biochemical tests such as nitrate reductase, heat stable catalase and sensitivity to PNB [24, 25].

### DNA extraction

DNA extraction was carried out from the processed clinical specimens by CTAB-chloroform method [26]. The concentration of DNA was determined by measuring the optical density at 260 nm by Nanodrop2000 (Thermo scientific, US). DNA used for PCRs were diluted in Tris-EDTA buffer (pH-7.8) to final concentrations of less than 40 μg/ml to overcome the action of potential PCR inhibitors.

### Two step nested multiplex PCR standardization for the *IS6110*, *MTP40* and *32-kD*α*-antigen* encoding gene sequences

Two step nested multiplex PCR assay was standardized and was found to have quantitative sensitivity to detect the DNA equivalent to 1-2 organisms. It tested positive with standard strain of *M. tuberculosis*, H37Rv. In each independent nMPCR assay, test results were compared with the results for one positive and one negative control. We used the reference strain H37Rv *M. tuberculosis* as positive control and molecular grade water (no target DNA) as negative control. *M. tuberculosis* H37Rv and *M. smegmatis* standard strain had been obtained from JALMA, Agra, India.

The identification and differentiation of MTBC from NTM was carried out by using specific pair of primers reported by Portillo et al. [27] to amplify *IS6110*, *MTP40 & 32kD* α*-antigen* encoding gene sequences (Table 1) specific for *M. tuberculosis* complex, *M. tuberculosis* and non-tuberculous mycobacteria respectively in single step multiplex PCR. In nested multiplex PCR we have designed specific pair of primers for amplification of *IS6110*, *MTP40 & 32kD* α*-antigen* encoding gene sequences which gave band sizes of 500 bp, 342 bp & 413 bp respectively (Table 2).

**Table 1:** Sequences of primers used in the detection of *Mycobacterium* spp. in single step multiplex PCR assay

Genes	Primers	Sequence (5’-3’)	Size (bp)	Reference
*MTP40* ^a^	MTB F	CGGCAACGCGCCGTCGGTGG	396	Herrera E. A. 1996 [15]
	MTB R	CCCCCCACGGCACCGCCGGG		
*IS6110* ^b^	MTBC F	CGGAGACGGTGCGTAAGTGG	984	Wojciech, 1992 [39]
	MTBC R	GATGGACCGCCAGGGCTTGC		
*32kD* α*-antigen* ^c^	NTM F	TTCCTGACCAGCGAGCTGCCG	506	Ohara et al., 1993 [40]
	NTM R	CCCCAGTACTCCCAGCTGTGC		

^a^specific for *Mycobacterium tuberculosis*

^b^specific for *Mycobacterium tuberculosis* complex

^c^specific for non-tuberculous mycobacterium

**Table 2:** Sequences of primers used in nested multiplex PCR for the detection of *Mycobacterium spp.*

Genes	Primers	Sequence (5’-3’)	Size (bp)	Reference (Primers)
*nMTP40* ^a^	MTB2F	CGTTCGGGATGCACTGCG	342	In this study
	MTB2R	CACCCGGCGAATTCGTCAC		
*nIS6110* ^b^	MTBC2F	CGATCGCCCCATCGACCTACT	500	In this study
	MTBC2R	GGTCGAGTACGCCTTCTTGT		
*n32kD* α*-antigen* ^c^	NTM2F	CACCCGCAGTTCATCTA	413	In this study
	NTM2R	CGTTGTAGGCGTCCTGG		

*n* Primers used in nested PCR

^a^specific for *Mycobacterium tuberculosis*

^b^specific for *Mycobacterium tuberculosis* complex

^c^specific for non-tuberculous mycobacterium

The first round PCR master mix was prepared by mixing 24 μl of a previously mixed reaction mixture [containing 2.5 μl of 10X reaction buffer (GeNei, Banglore, India), 2.5 μl of 200 μM concentrations of each of the deoxynucleoside triphosphates (dNTPs) (GeNei, Banglore, India), 0.1 μl of 5U *Taq* DNA Polymerase (GeNei, Banglore, India) and 1 μl of the each oligonucleotide primers (10 pmol) (GeNei, Banglore, India)], 5 μl of the DNA template and added milli Q to create a total volume of 25 μl. The first-round amplification was carried out in a thermocycler (T-100™-Bio-Rad) under the following conditions: initial denaturation at 95 °C for 3 min, 35 cycles of 94 °C for 1 min, 65 °C for 1 min and 72 °C for 50 s and a final extension at 72 °C for 7 min. In nested multiplex PCR, reaction mixture was same as that in first round PCR, except it contained 15 pmol of second round primers (Table 2) and 2 μl of 1:6 diluted product of the primary cycle as DNA template. The amplification conditions were identical to that of first round PCR. The amplified products of both cycles were analyzed by agarose gel electrophoresis (GeNei, Banglore, India) on 2 % agarose gel (GeNei, Banglore, India) using 5 μl of amplified products. The gels were stained with ethidium bromide (0.5 μg/ml) and bands were visualized under UV light.

### Statistical analysis

Sensitivity, specificity, positive predictive value and negative predictive value of the nMPCR were evaluated by using online MedCalc and compared with single step PCR, microbiological tests and CRS.

### Ethics statement

This study has been ethically approved by the ethical committee of the Institute of Medical Sciences, Banaras Hindu University, Varanasi, India.

## Results

### Clinical data

Out of 600 patients 223 (37.2 %) were females and 377 (62.8 %) were males. The participants ranged from 2 to 90 years (median age 26.3 ± 16.8). Most of the patients [267/600; (44.5 %) were in 20–40 years age group.

### Patient categories

**Fig. 1:**
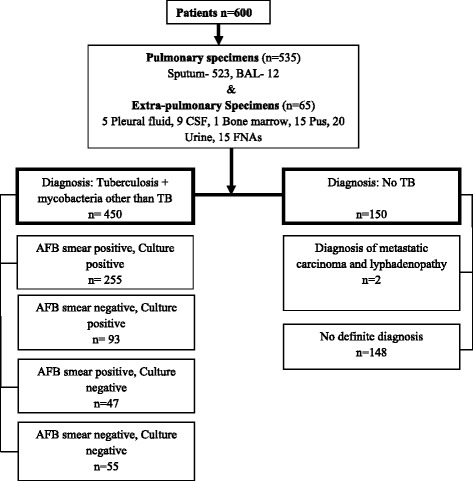
Flow chart of the participants included and analyzed in this study

A total of 600 patients were considered in this study. Out of these participants, 348 (58.0 %) were culture positive “definitive TB” cases [255 (42.5 %) being smear positive and 93 (15.5 %) being smear negative]; 47 (7.8 %) were clinically, radiologically, and/or cytologically positive, suggestive of “probable TB” cases; 55 (9.2 %) were only clinically positive and responded to ATT, suggestive of “possible TB” cases; and 150 (25 %) patients had no evidence of TB and were “non-TB” cases. Details of study participations are depicted in Fig. 1 of 450 specimens, 92.2 % (*n* = 415) pulmonary and 7.8 % (*n* = 35) extra-pulmonary specimens were able to fit the CRS criteria as reference.

### Culture and microscopy

Out of 535 pulmonary samples, 53.6 % (*n* = 287) were positive for AFB bacilli whereas 61.5 % (*n* = 329) samples were positive for MTB culture. Of the 65 extra-pulmonary specimens 3.1 % (*n* = 2) pleural fluid, 4.6 % (*n* = 3) CSF, 9.2 % (*n* = 6) pus and 6.1 % (*n* = 4) FNAs were positive by Ziehl-Neelsen staining, while 4.6 % (*n* = 3) pleural fluid, 6.1 % (*n* = 4) CSF, 1.5 % (*n* = 1) bone marrow, 10.8 % (*n* = 7) pus and 6.1 % (*n* = 4) FNAs were culture positive (Table 3). From 600 specimens, 50.3 % (302/600) were AFB smear positive, 344 (57.3 %) were *M. tuberculosis* culture positive, 4 (0.7 %) were NTM positive and 241 (40.2 %) were culture negative. Eleven (1.8 %) were contaminated. Finally 348 specimens were mycobacteria culture positive which was considered in the final analysis.

**Table 3:** Comparison of smear, culture, single step mPCR and nMPCR for detection of *Mycobacterium spp.* in clinical samples

Nature of samples	No. of cases	CRS positive cases	Z-N smear (%)	Culture (%)	Single step mPCR (%)	nMPCR (%)	*IS6110 & MTP40* (%)	MTP40 & *32kD *α*-antigen*	Only *IS6110* (%)	Only *MTP40* (%)	Only *32kD* α*-antigen* (%)	*IS6110* + *MTP40*+ *32kD* α*-antigen*
Pulmonary samples	535	415 (77.6)	287 (53.6)	329 (61.5)	345 (64.5)	403 (75.3)	373 (69.7)	6 (1.1)	5 (0.9)	7 (1.3)	9 (1.7)	3 (0.6)
Sputum	523	409 (78.2)	283 (53.3)	323 (61.7)	337 (64.4)	394 (75.3)	365 (69.8)	6 (1.1)	4 (0.8)	7 (1.3)	9 (1.7)	3 (0.6)
BAL	12	6 (50)	4 (33.3)	6 (50)	8 (66.6)	9 (75)	8 (66.6)	0	1 (0.2)	0	0	0
Extra-pulmonary samples	65	35 (53.8)	15 (23.1)	19 (29.2)	20 (30.8)	32 (49.2)	22 (33.8)	1 (1.5)	2 (3.0)	2 (3.0)	4 (6.1)	1 (1.5)
Pleural Fluid	5	3 (60)	2 (40)	3 (60)	3 (60)	3 (60)	3 (60)	1	0	0	0	0
CSF	9	6 (66.7)	3 (33.3)	4 (44.4)	4 (44.4)	5 (55.5)	4 (44.4)	0	0	1 (11.1)	0	0
Bone marrow	1	1 (100)	0	1 (100)	1 (100)	1 (100)	1 (100)	0	0	0	0	0
Pus	15	12 (80)	6 (40)	7 (46.7)	6 (40)	12 (80)	7 (46.7)	1 (6.7)	1 (6.7)	1 (6.7)	1 (6.7)	1 (6.7)
Urine	20	4 (20)	0	0	1 (5)	3 (15)	3 (15)	0	0	0	0	0
FNAs	15	9 (60)	4 (26.7)	4 (26.7)	5 (33.3)	8 (53.3)	4 (26.7)	0	1 (6.7)	0	3 (20)	0
Total		450	302 (67.1)	348 (77.3)	365 (81.1)	435 (96.7)	395 (87.8)	7 (1.5)	7 (1.5)	9 (2.0)	13 (2.9)	4 (1.0)
Control	150	0	-	-	-	2 (1.3)	2 (1.3)	-	-	-	-	-
Total	600	-	-	-	-	437 (72.8)	397 (66.2)	-	-	-	-	-

*BAL* bronchoalveolar lavage, *CSF* cerebrospinal fluid, *FNAs* fine needle aspirates, *mPCR* multiplex PCR, *nMPCR* nested multiplex PCR

### Evaluation of nested multiplex PCR

**Fig. 2:**
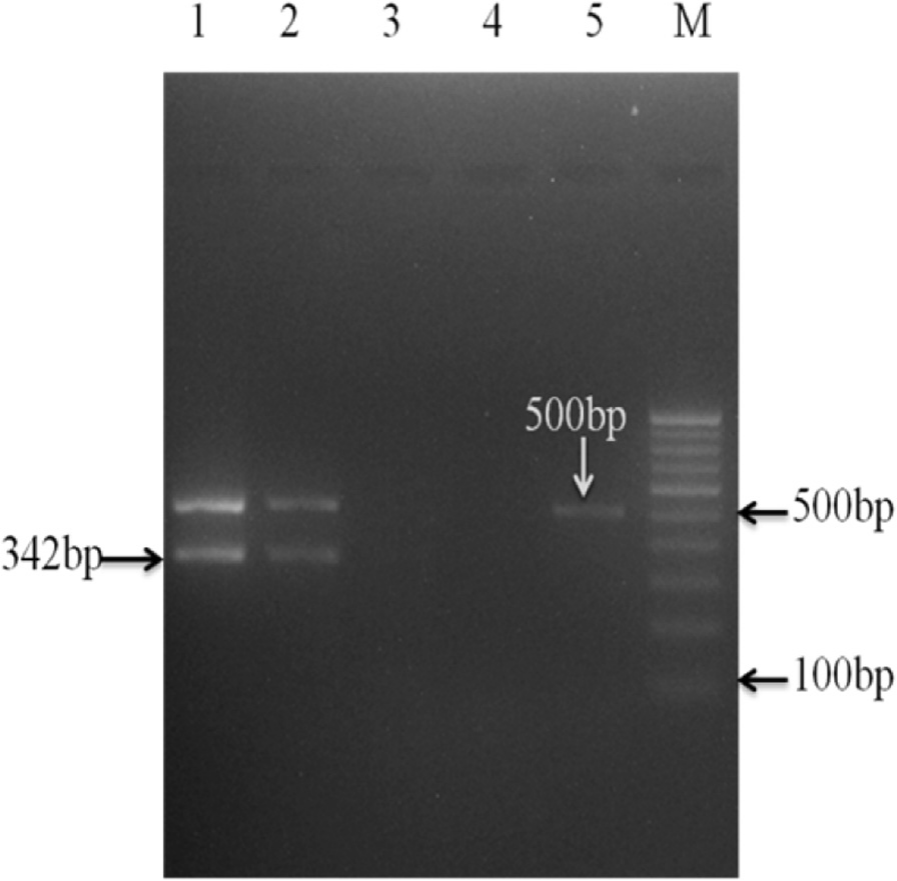
Agarose gel electrophoresis of nested multiplex PCR for the identification of *Mycobacterium tuberculosis* complex from clinical samples. Lane-1: positive control (H37Rv); lane-2: clinical samples positive for *IS6110* and *MTP40*; Lane-3- negative control (MQ water); Lane-4- negative clinical sample; lane-5- patient samples positive for only *IS6110*and negative for *MTP40*

**Fig. 3:**
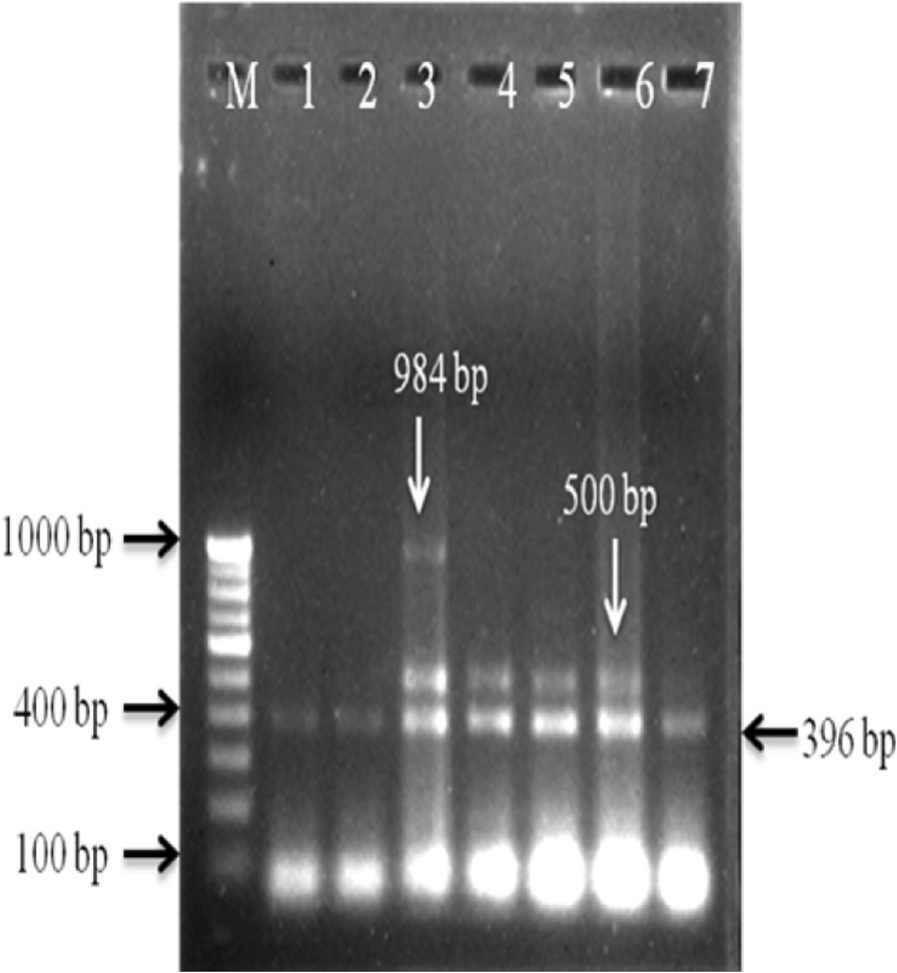
Agarose gel electrophoresis showing mixed infection in single step multiplex PCR. M- 100 bp DNA marker; lane-1, 2: clinical samples positive for *MTP40*; lane-3: clinical sample positive for *MTP40, IS6110* and *32kD alpha antigen* (Mixed infection); lane- 4, 5, 6 & 7: clinical samples positive for *MTP40 and IS6110*

The results obtained by nMPCR for pulmonary and extra-pulmonary specimens are shown in Table 3. On subjecting all the clinical specimens to nMPCR, 75.3 % (403/535) pulmonary and 49.2 % (32/65) extra-pulmonary samples were positive for either or all the target genes namely *IS6110*, *MTP40* and *32kD-*α *antigen*. The gel picture is shown in Fig. 2. Further, detection of TB increased from 60.8 % (365/600) by single step mPCR to 72.5 % (435/600) by nMPCR for clinical specimens. In addition, with regard to PCR target detection in the nested multiplex PCR protocol, 87.8 % [including 69.7 % (373/535) pulmonary and 33.8 % (22/65) extra-pulmonary specimens] of the 450 clinically confirmed and suspected TB cases showed both *IS6110* and *MTP40* amplified bands patterns which belongs to the MTBC and MTB. Further, 1.1 % (6/535) pulmonary and 1.5 % (1/65) extra-pulmonary (pus) samples were positive for *MTP40* and *32kD* α*-antigen* encoding gene (mixed infection) and 0.8 % (4/450) cases were positive for *32kD* α*-antigen* encoding gene, *IS6110* and *MTP40* (mixed infection) (Fig. 3). No individual sample was established by culture to harbour both *M. tuberculosis* and non tubercular mycobacteria, as was seen with samples examined by the nMPCR assay. Among 450 TB cases, only *IS6110* band pattern was detected in 1.5 % (*n* = 7) samples including 4 sputum, 1 BAL, 1 pus and 1 FNA and only *MTP40* band was detected in 2.0 % (*n* = 9) samples including 7 sputum, 1 CSF and 1 pus. Only *32kD* α*-antigen* encoding gene band pattern was obtained in 2.9 % (13/450) specimens.

Of the 150 samples (control), from those patients without mycobacterial infections, 98.7 % (*n* = 148) samples were found to be nested multiplex PCR negative, as expected; whereas, remaining 1.3 % (*n* = 2) samples were positive for both *IS6110* and *MTP40* band patterns (Table 3).

### Diagnostic association of nMPCR results with composite reference standard criteria

Table 4 represent the association of nMPCR and single step mPCR with CRS in clinical specimens. Among the S + C+ specimens (Group A), single step mPCR and nMPCR assay detected mycobacterial DNA in 92.9 % (237/255) and 100 % (255/255) of specimens respectively. Among the S-C+ specimens (Group A), 77.4 % (72/93) specimens were single step mPCR positive and 98.9 % (92/93) were positive for nMPCR assay. From this group 8.6 % (22/255) and 15.3 % (39/255) of the samples were single step mPCR positive for only *MTP40* and *IS6110* band patterns but in nMPCR only 3.5 % (9/255) and 2.3 % (6/255) of the specimens were positive for only *MTP40* and *IS6110* respectively (Additional file 1).

**Table 4:** Comparison of conventional procedures with single step mPCR and nested multiplex PCR for detection of *Mycobacterium spp.* in different groups of clinical specimens

Type	Study group	No. of patients (*n* = 600)	Single-step mPCR (%)	Nested multiplex PCR (%)
Group A	Definitive TB group (*N* = 348)			
	AFB smear + ve Culture + ve	255 (42.5)	237 (92.9)	255 (100)
	AFB smear –ve Culture + ve	93 (15.5)	72 (77.4)	92 (98.9)
Group B	Probable TB group (*N* = 47)			
	AFB smear + ve Culture –ve	47 (7.8)	31 (66.0)	45 (95.7)
Group C	Possible TB group (*N* = 55)			
	AFB smear -ve Culture –ve	55 (9.2)	25 (45.4)	43 (78.2)
Group D	Non-TB group (*N* = 150)	150 (25)	0	2 (1.3)
Total		600	365 (60.8)	437 (72.8)

*+ve* positive, *-ve* negative

Out of 47 S + C- specimens (Group B) 66.0 % (*n* = 31) were single step mPCR positive and 95.7 % (*n* = 45) were positive for nMPCR. On the other hand, 45.4 % (25/55) and 78.2 % (43/55) of the S-C- specimens were positive for single step PCR and nMPCR assay respectively (Table 4). There details for specific band patterns are shown in additional file 1. Finally, out of 150 samples taken from patients without mycobacterial infection, 148 (included in Group D) were found to be negative for both single step mPCR and nMPCR whereas, remaining 2 (1.3 %) samples were positive for nMPCR assay but negative in single step mPCR.

There were 9 samples in group A which showed absence of *IS6110* and presence of *MTP40* band pattern in nMPCR assay indicating presence of *M. tuberculosis* strains lacking *IS6110* gene sequence. Besides, some specimens from group A and B showed presence of members of MTBC other than *M. tuberculosis* (Additional file 1).

### Sensitivity and specificity

Performance of all tests using CRS as a gold standard for all clinical specimens are presented in Table 5. As can be seen, nMPCR test have higher sensitivity than smear, culture and single step mPCR in different clinical samples. The sensitivities of smear, culture, single step PCR, nMPCR tests and CRS were found to be 69.2, 79.3, 83.1, 97.1 and 100 % for PTB and 42.9, 54.3, 57.1, 91.4 and 100 % for EPTB cases.

**Table 5:** Sensitivity and specificity of conventional methods, nMPCR assay and CRS with respect to different specimens

Different testes	Test results	TB group	Control (*n* = 150)	Sensitivity (%)	Specificity (%)	PPV (%)	NPV (%)	CI
Pulmonary TB (*n* = 535)								
AFB smear	Positive Negative	287 128	0 120	69.2	100	100	48.4	64.5 % – 73.6 %
Culture	Positive Negative	329 86	0 120	79.3	100	100	58.2	75.0 % – 83.1 %
Single step mPCR	Positive Negative	345 70	0 120	83.1	100	100	63.2	79.2 % – 86.6 %
nMPCR	Positive Negative	403 12	0 120	97.1	100	100	90.9	95.0 % – 98.5 %
CRS	Positive Negative	415 0	0 120	100	100	100	100	99.1 % –100.0 %
Extra-pulmonary TB (*n* = 65)								
Z-N smear	Positive Negative	15 20	- 30	42.9	100	100	60.0	26.3 % – 60.6 %
Culture	Positive Negative	19 16	- 30	54.3	100	100	65.2	36.6 % – 71.2 %
Single step mPCR	Positive Negative	20 15	- 30	57.1	100	100	66.7	39.3 % – 73.7 %
nMPCR	Positive Negative	32 3	2 28	91.4	93.3	94.1	90.3	76.9 % – 98.2 %
CRS	Positive Negative	35 0	0 30	100	100	100	100	90.0 % to 100.0 %

*PPV* positive predictive value, *NPV* negative predictive value, *TB group* TB suspected patients, *mPCR* multiplex PCR, *nMPCR* nested multiplex PCR, *CRS* composite reference standard

## Discussion

Early identification of the infecting microorganism is necessary for patient management. In the case of MTB infection, rapid diagnosis and identification is a major factor for starting anti-TB drug therapy and to control the dissemination of MTB bacilli from person to person. Further, it is necessary to differentiate MTBC members from NTM for proper management of tuberculosis. The precise and early diagnosis of extra-pulmonary TB is challenging due to the paucibacillary nature of the specimens and requiring long incubation time for growth of tubercle bacilli. This long delay without positive or negative microbiological results could be shortened by the use of amplification techniques. However, these methods have relatively low sensitivity when applied directly to extra-pulmonary samples [28].

Therefore, we evaluated the utility of nested multiplex PCR protocol for paucibacillary pulmonary and extra-pulmonary specimens. The test detected mycobacterial DNA in 99.7 % (347/348) specimens of ‘confirmed TB’ cases, including 26.4 % (92/348) of smear negative TB cases. It was also observed that nMPCR protocol detected mycobacterial DNA in 95.7 % (45/47) of the samples from ‘probable TB’ cases, whose culture were negative but who had positive radiological tests and/or positive cytolopathology reports. Whereas, 78.2 % (43/55) of the samples, whose smear and culture were negative from ‘possible TB’ cases but patients under ATT shedding non-cultivable bacteria also detected by nMPCR. It has been reported that nested PCR could detect those mycobacteria that are non- cultivable [29]. One hundred forty eight samples (non-TB cases) from patients without mycobacterial infections were negative for CRS criteria and nMPCR assay. It means these patients were considered as truly negative for TB infection. Whereas, remaining two samples from patients with metastatic carcinoma and lymphadenopathy, who had no clinical symptoms of TB may be false- positive result by nMPCR. The immunosuppressive nature of a malignant lesion may have been capable of reactivating a latent tubercular infection; hence, there may be coexistence of malignancy and tuberculosis.

A previous study has reported highest PCR positivity rates for pulmonary (77 %) and extra-pulmonary (11 %) samples using *IS6110* as a specific target sequence [30]. However in some parts of the world, few strains do not possess the *IS6110* target sequence in their genome, which may cause a false negative result and thereby decrease the sensitivity of the assay [31, 32]. Recently, one study has reported 41.6 % and 36 % PCR positivity by multiplex PCR targeting *MTP40* and *IS6110* respectively [11]. Although, some studies have standardized the nested PCR assay to detect even single bacillus in clinical samples which have increased the sensitivity for detection of MTB, nested PCR using single target alone could not differentiate members of MTBC from non-tubercular mycobacteria [33, 34]. Therefore, in this study we have used more than two target sequences in nMPCR assay, which increased the positivity (72.8 %) for detection of mycobacterial DNA. Mixed infection of NTM and *Mycobacterium tuberculosis* was found in 0.2 % cases by Aliyu et al. [7]. We have found that 2.4 % (11/450) cases are shown mixed infection of NTM and MTBC. Higher positivity of nMPCR might be due to its ability to detect less amount of target DNA (1 bacilli/μl) directly from clinical samples even in the presence of relatively large amount of human DNA [33] and may also be due to selection of multiple target sequences included *MTP40*, *IS6110* and *32kD-*α *antigen* encoding gene in a two step reaction.

The sensitivity of nMPCR was observed to be 97.1 % and 91.4 % with CRS criteria for pulmonary and extra-pulmonary samples respectively which was greater than 80.2 % [17] and 91 % [18] sensitivity reported in the previous studies using multiplex PCR and nested PCR respectively. Specificity of nMPCR assay was observed to be 100 % and 93.9 % respectively for pulmonary and extra-pulmonary TB cases with CRS criteria which were comparable with that of 94.4 % [17] and 91 % [18] reported in the previous studies. In this study we have used more than two targets in nested multiplex PCR protocol due to which sensitivity increased from 83.1 to 97.1 % for pulmonary and from 57.1 to 91.4 % for the EPTB diagnosis. In group D two specimens were considered as false negative by CRS criteria but using nMPCR assay it becomes positive. Therefore specificity was found to be 93.3 % for nMPCR assay in extra-pulmonary TB cases.

The low sensitivity of culture (79.3 % and 54.3 %) and single step mPCR assay (83.1 % and 57.1 %) for PTB and EPTB cases in comparison with that of nMPCR test (97.1 % and 91.4 %) may be due to the fact that 86 % (88/102) of the culture negative, nMPCR positive patients were on ATT for various time periods; unequal distribution of mycobacteria in paucibacillary respiratory specimens, low number of bacterial load in extra-pulmonary specimens and non-uniform dispersion of microorganisms in clinical specimens causes clumping of the microorganisms which is the most common problem with mycobacteria [34, 35]. Moreover, nMPCR can detect a single bacillus present in clinical specimens than culture and single step mPCR [33, 36].

The limitation with nMPCR assay is that it is not able to differentiate live from dead organisms. Hence, this method should be recommended only for screening of new cases but not for monitoring of patients in treatment. Liquid culture (MIGIT 960) could have increased the sensitivity as it is known to be 10 % more sensitive [37]. Also using 4 % NaOH for decontamination is a limitation of this study as it is known to be a harsh decontaminant. The advantage of nested multiplex PCR is that it improves the sensitivity and specificity using signature nucleotides for the detection and differentiation of MTBC from NTM in clinical specimens that were even smear and culture negative. To the best of our knowledge, although nested multiplex PCR assay has been used for the diagnosis of pulmonary TB, there is only one report of it being used for EPTB cases [38]. It is obvious that further evaluation is needed in order to improve this nested multiplex PCR protocol for routine diagnosis.

## Conclusions

Our study advocates that nested multiplex PCR is a highly sensitive and specific technique for the diagnosis of pulmonary and extra-pulmonary TB which is often not only missed by conventional methods but also by single-step multiplex PCR due to low bacillary load, resulting in an unacceptable delay in initiation of therapy. Further, it can also be used to detect samples with *M. tuberculosis* strains lacking *IS6110*. This study also concludes that the CRS criteria were better for assessing the diagnostic accuracy of nested multiplex PCR assay.

## Additional file

Additional file 1: Comparison of conventional procedures with single-step & nested multiplex PCR for detection of M. tuberculosis complex in different groups of patients. (DOCX 15 kb)

## Abbreviations

AFB: acid fast bacilli
ATT: anti-tubercular therapy
BAL: bronchoalveolar Lavage
CRS: composite reference standard
CSF: cerebrospinal fluid
EPTB: extra-pulmonary tuberculosis
FNAC: fine needle aspirate cytology
FNAs: fine needle aspirates
LJ: lowenstein jensen
mPCR: multiplex PCR
MTB: *Mycobacterium tuberculosis*
MTBC: *Mycobacterium tuberculosis* complex
NAAT: nucleic acid amplification technique
nMPCR: nested multiplex PCR
nPCR: nested polymerase chain reaction
NTM: nontuberculous mycobacteria
PCR: polymerase chain reaction
PTB: pulmonary tuberculosis
S + C-: smear positive culture negative
S + C+: AFB smear positive and culture positive
S-C-: smear negative and culture negative
S-C+: smear negative and culture positive
