# Supplementary material for: Differentiation of Mycobacterium tuberculosis complex from non-tubercular mycobacteria by nested multiplex PCR targeting IS6110, MTP40 and 32kD alpha antigen encoding gene fragments
Source: BMC Infect Dis. 2016 Mar 12;16:123. doi: 10.1186/s12879-016-1450-1 (PMC4788904; doi:10.1186/s12879-016-1450-1)
Supplement: Additional file 1: — Comparison of conventional procedures with single-step & nested multiplex PCR for detection of M. tuberculosis complex in different groups of patients. (DOCX 15 kb) [file 12879_2016_1450_MOESM1_ESM.docx]

**Additional File 1. Comparison of conventional procedures with single-step & nested multiplex PCR for detection of *M. tuberculosis* complex in different groups of patients**

| **Type** | **Study Group** | **Total no.**  **(n=600)** | **Single-step PCR** | | | | | | **Nested multiplex PCR** | | | | | |
| --- | --- | --- | --- | --- | --- | --- | --- | --- | --- | --- | --- | --- | --- | --- |
|  |  |  | ***IS6110 & MTP40***  **n (%)** | **Only *MTP40***  **n (%)** | **Only *IS6110***  **n (%)** | ***32kD α-antigen* & *MTP40***  **n (%)** | **Only**  ***32kD α-antigen***  **n (%)** | ***IS6110+***  ***MTP40+***  ***32kD α-antigen* n (%)** | ***IS6110 & MTP40***  **n (%)** | **Only *MTP40***  **n (%)** | **Only *IS6110***  **n (%)** | ***32kD α-antigen* & *MTP40***  **n (%)** | **Only**  ***32kD α-antigen***  **n (%)** | ***IS6110+MTP40+***  ***32kD α-antigen***  **n (%)** |
| **Group A** | **Definitive TB group**  S+C+  S-C+ | 255 (42.5)  93 (15.5) | 166 (65.1)  70 (75.3) | 22 (8.6)  0 | 39 (15.3)  0 | 4 (1.6)  0 | 5 (2.0)  2 (2.1) | 1 (0.4)  0 | 228 (89.4)  86 (92.5) | 9 (3.5)  0 | 6 (2.3)  0 | 5 (2.0)  2 (2.1) | 6 (2.3)  3 (3.2) | 1 (0.4)  1 (1.1) |
| **Group B** | **Probable TB group**  S+C- | 47 (7.8) | 30 (63.8) | 0 | 0 | 0 | 1 (2.1) | 0 | 42 (89.3) | 0 | 0 | 0 | 2 (4.2) | 1 (2.1) |
| **Group C** | **Possible group**  S-C- | 55 (9.2) | 22 (40) | 0 | 0 | 0 | 2 (3.6) | 1 (1.8) | 39 (71.0) | 0 | 1 (1.8) | 0 | 2 (3.6) | 1 (1.8) |
| **Group D** | **Non-TB group** | 150 | 0 | 0 | 0 | 0 | 0 | 0 | 2 (1.3)* | 0 | 0 | 0 | 0 | 0 |
| **Total** |  | **600** | **288 (48.0)** | **22 (3.7)** | **39 (6.5)** | **4 (0.7)** | **10 (1.7)** | **2 (0.3)** | **397 (66.2)** | **9 (1.5)** | **7 (1.2)** | **7 (1.2)** | **13 (2.2)** | **4 (0.7)** |

*Possible false positive results by the *IS6110* gene fragment.
